# miR-143-3p Promotes T_SCM_ Differentiation and Inhibits Progressive T Cell Differentiation via Inhibiting ABL2 and PAG1

**DOI:** 10.3390/genes16040466

**Published:** 2025-04-19

**Authors:** Wenkai Shi, Jieming Hu, Hongqiong Wang, Huishan Zhong, Wenfeng Zhang, Jinquan Wang, Hongwei Shao, Han Shen, Huaben Bo, Changli Tao, Fenglin Wu

**Affiliations:** School of Life Sciences and Biopharmaceutics, Guangdong Pharmaceutical University, Guangzhou 510006, China; 18157960576@163.com (W.S.); jieminghu519000@163.com (J.H.); 13380874632@163.com (H.W.); 18344391326@163.com (H.Z.); zwfsnowdream@126.com (W.Z.); wangjinquan@gdpu.edu.cn (J.W.); shaohw2000@163.com (H.S.); shenhanbc@163.com (H.S.); ben1702@126.com (H.B.); taochangli@126.com (C.T.)

**Keywords:** T cell, microRNA, progressive differentiation

## Abstract

Background: Adoptive cell therapy (ACT), including CAR-T and TCR-T therapies, shows promise for cancer treatment, depending on infused T cell expansion, persistence and activity. We previously characterized four T-cell subsets (T_N_, T_SCM_, T_CM_ and T_EM_) and their miRNA profiles. Objectives: This study investigates miR-143-3p’s role in T cell differentiation. Methods: Using qPCR, we analyzed miR-143-3p expression. Target genes were validated by dual-luciferase assays. Functional assays assessed differentiation markers, proliferation, apoptosis and cytokine secretion. Results: miR-143-3p was upregulated in early-differentiated T_SCM_ but downregulated during progression. We confirmed *ABL2* and *PAG1* as direct targets suppressed by miR-143-3p. Overexpression increased early markers (LEF1, CCR7 and CD62L) while decreasing late markers (EOMES, KLRG1 and CD45RO). It also enhanced proliferation, reduced apoptosis and suppressed cytokine secretion. Conclusions: miR-143-3p promotes T_SCM_ differentiation and inhibits progressive differentiation by targeting ABL2/PAG1, suggesting new ACT optimization strategies.

## 1. Introduction

In recent years, adoptive cell therapy (ACT), demonstrated through antibody-based chimeric antigen receptor (CAR)-T cell intervention and T cell receptor (TCR)-modified T cell treatment, has been developed as an effective strategy for cancer care. Chimeric antigen receptor (CAR) T cell immunotherapy has been clinically approved for the management of diffuse large B-cell lymphoma (DLBCL), B-cell acute lymphoblastic leukemia (B-ALL) and recurrent or refractory multiple myeloma [1,2]. Clinical trials have also been conducted on solid tumors, such as gastrointestinal cancers, prostate cancer and melanoma [3,4]. Meanwhile, TCR-T therapy has been approved for the treatment of unresectable and metastatic uveal melanoma, and clinical trials are underway for solid tumors, including cervical cancer, head and neck squamous cell carcinoma, lung cancer and melanoma [5].

However, ACT still faces several challenges that need to be addressed. One of the key difficulties is that T cells used for genetic modification and reinfusion require in vitro activation, which often induces them into terminally differentiated phenotypes, leading to a state of dysfunction. Studies have shown that using T cells that possess and maintain less differentiated phenotypes (such as memory and precursor T cells) for ACT can significantly enhance antitumor efficacy and improve patient outcomes [6,7]. The progressive differentiation model of T cells provides theoretical support for explaining this phenomenon [8]. This model posits that T cell differentiation follows a progressive pattern: T_N_ → T_SCM_ → T_CM_ → T_EM_ → T_EFF_. Less differentiated T cell populations (including naïve and memory subsets) demonstrate enhanced proliferative capacity, prolonged persistence, and greater potential to generate effector progeny capable of mediating antitumor immunity [9].

Researchers are working to increase the proportion of less differentiated T cells in ACT or to inhibit the progressive differentiation of T cells to enhance their antitumor efficacy. Various approaches have been employed to obtain less differentiated T cells, including the use of optimized culture conditions (e.g., replacing IL-2 with IL-7, IL-15 and IL-21 to promote T cell differentiation toward a memory phenotype), modulation of CAR signaling and expression [10], epigenetic regulation (e.g., DNA methylation and histone modification) [11], control of T cell differentiation [12] and modulation of T cell metabolism (e.g., inhibition of glycolysis) [13].

Our previous studies have demonstrated that overexpressing characteristic transcription factors of early-stage T cells (T_N_, T_SCM_) in terminally differentiated T_EFF_ cells can induce their dedifferentiation. In addition to transcription factors, emerging studies have established the pivotal regulatory roles of microRNAs in T lymphocyte differentiation processes, cellular expansion and programmed cell death. These small non-coding RNA molecules, typically 19–22 nucleotides in length, function through complementary base pairing with the 3′ untranslated regions of target mRNAs to post-transcriptionally repress gene expression [14], ultimately influencing cell fate and differentiation. For example, overexpression of miR-181a results in significant suppression of a broad spectrum of phosphatases at both transcriptional and translational levels, thereby modulating the intensity of TCR signaling in T cells [15]. miR-34a activates dendritic cell-mediated innate immune responses and increases the infiltration of CD8+ T lymphocytes in tumors [16]. Inhibition of miR-15/16 enhances cytotoxic T cell activation and memory cell formation [17].

Using advanced cell sorting techniques, we previously isolated four discrete T cell populations (T_N_, T_SCM_, T_CM_ and T_EM_) along the differentiation continuum, followed by systematic miRNA expression analysis using next-generation sequencing platforms. The present investigation specifically examined differential miRNA expression between T_SCM_—the initial differentiation phase post-T cell activation—and other subsets. Our data demonstrate minimal miR-143-3p expression in naïve T cells (T_N_). Following activation, miR-143-3p expression increases significantly, showing particularly elevated levels in T_SCM_. Progressive T cell differentiation leads to a gradual reduction in miR-143-3p expression. Through target prediction and functional annotation using KEGG and GO databases, we identified that miR-143-3p potentially modulates T cell differentiation via transcription factor regulation. Luciferase reporter assays verified direct targeting and suppression of *ABL2* and *PAG1* by miR-143-3p in T cells. Ectopic expression of miR-143-3p enhances T_SCM_ differentiation while restraining progressive T cell differentiation.

## 2. Materials and Methods

### 2.1. Screening of Differentially Expressed miRNAs

Based on our previous study [18], miRNA expression profiles were obtained and normalized using the Transcripts Per Million (TPM) method [19]. The four T cell subsets—naïve T cells (T_N_), stem cell-like memory T cells (T_SCM_), central memory T cells (T_CM_), and effector memory T cells (T_EM_)—were divided into three groups to analyze differentially expressed miRNAs in T_SCM_ during progressive T cell differentiation. Pairwise comparisons were performed between adjacent groups (T_SCM_-T_N_, T_SCM_-T_CM_ and T_SCM_-T_EM_).Gene expression differential analysis was performed utilizing the limma package within the R statistical environment [20]. The screening criteria for miRNAs were set as a fold change (FC) ≥ 2 and a false discovery rate (FDR) ≤ 0.05, with a threshold of log2FC > 1 or <−1. The *p*-values were adjusted using the Benjamini and Hochberg method, and differentially expressed miRNAs were visualized using the pheatmap package (version 1.0.12) to generate clustered heatmaps.

$$TPM=\frac{miR\;readscounts\times 1,000,000}{Librarysize}$$

### 2.2. miRNA Target Gene Prediction and Visualization Network Construction

Potential target genes were identified through miRWalk 3.0 [21]. Candidate targets were defined as genes predicted by a minimum of two out of three prediction databases: TargetScan [22], miRDB [23] and miRTarBase [24]. The interaction network between miRNAs and their targets was constructed using Cytoscape (v3.2.1) [25]. Functional enrichment analysis, including Gene Ontology (GO) [26] and KEGG pathway [27] analysis, was carried out for miRNAs targeting over five genes using clusterProfiler (v2.4.3). For computationally predicted miR-143-3p targets lacking experimental validation, GeneTrail2 [28] was employed with a significance cutoff of *p* < 0.05. Transcription factor binding analysis for selected miR-143-3p targets was conducted using Cytoscape’s ClueGO plugin [29].

### 2.3. T Cell Activation

Lymphocytes were adjusted to 1–2 × 10^6^ cells/mL in RPMI-1640 medium (Gibco, Grand Island, NY, USA) containing 10% FBS (Gibco, Grand Island, NY, USA). For activation, cells were treated with 2 µg/mL anti-CD3 (OKT3, eBioscience, San Diego, CA, USA), 1 µg/mL anti-CD28 (CD28.2, eBioscience, USA) and 300 IU/mL IL-2 (PeproTech, Cranbury, NJ, USA) for 48–60 h. T cells were transfected with 50 nM miR-143 mimic/inhibitor or corresponding NCs using Lipofectamine 3000 (Invitrogen, Carlsbad, CA, USA).

### 2.4. Dual-Luciferase Reporter Assay for miR-143-3p Target Genes

Wild-type reporter plasmids were created through ligation of 200-bp genomic fragments encompassing the predicted miR-143-3p binding regions in *ABL2* and *PAG1* 3′UTRs into psiCHECK-2 dual-luciferase vectors. Mutant versions were created via PCR-mediated site-directed mutagenesis (A/T to G/C) using wild-type plasmids as templates. For miRNA^POS^ construction, the antisense sequence of miR-143-3p was inserted into psiCHECK-2. HEK-293T cells were plated in 24-well plates (2 × 10^5^ cells/well) 24 h prior to transfection. Using Lipofectamine 3000, the wild-type/mutant plasmids, miRNA^POS^ or empty vector were co-transfected with miR-143 mimics or NCs into HEK-293T cells. Luciferase activity was assessed 48 h post-transfection with a dual-luciferase assay kit (Yeasen, Shanghai, China).

### 2.5. RNA Extraction and Quantitative Real-Time PCR

Total RNA isolation was carried out with the EZ-press RNA Purification Kit (EZBioscience). The Color Reverse Transcription Kit (EZBioscience) was employed for cDNA generation. qPCR analysis was performed using 2 × SYBR Green qPCR Master Mix (EZBioscience) on a LightCycler^®^ 480 Instrument II (Roche). Gene expression levels were determined via the 2^(−ΔΔCt)^ method, with primer sequences provided in Table 1.

### 2.6. Western Blot

Protein isolation was conducted using a RIPA lysis buffer (Beyotime, Shanghai, China) supplemented with protease inhibitors. Protein quantification was conducted using a BCA protein assay kit (Beyotime). Samples were electrophoresed on 2–20% SDS-PAGE gels at 80 V and transferred onto PVDF membranes (Biosharp, Hefei, China). After blocking with 5% skim milk for 2 h at RT, membranes were probed with primary antibodies against GAPDH (Proteintech, Wuhan, China, 1:50,000), ABL2 (Proteintech, 1:6000) and PAG1 (Proteintech, 1:6000) at 4 °C overnight. HRP-conjugated goat anti-mouse secondary antibody (Proteintech, 1:6000) was then applied for 1 h at RT. Protein bands were visualized using ECL substrate (GE Healthcare, Milwaukee, WI, USA) and quantified with ImageJ software (Version 2.15.0).

### 2.7. Flow Cytometry

Cell surface markers (CD3, CCR7, CD45RO, CD62L and CD95; eBioscience) were detected witht antibody staining followed by analysis on a Beckman Gallios flow cytometer. Acquired data were processed using FlowJo software (version 10).

### 2.8. Apoptosis and Proliferation Assays

For proliferation assays, single-cell suspensions (5 × 10^5^ cells/well) were plated in 96-well plates. Following the addition of CCK-8 reagent (YEASEN #40203ES76; 10 µL/well), plates were maintained at 37 °C/5% CO_2_ for 4 h before optical density measurement at 450 nm (Varioskan Flash, Thermo, Waltham, MA, USA). Apoptosis evaluation was performed after a 5-day culture with IL-2 (50 U/mL) using Annexin V-FITC/PI staining (MultiSciences, Hangzhou, China), with subsequent quantification of viable, apoptotic and necrotic populations by FlowJo V10 analysis.

### 2.9. Enzyme-Linked Immunosorbent Assay (ELISA)

Cell culture supernatants were collected and centrifuged at room temperature. Concentrations of TNF-α and IFN-γ were measured using Human TNF-α and IFN-γ ELISA Kits (MultiSciences), respectively.

### 2.10. Statistical Analysis

Three independent replicates were conducted for each experiment. Results are expressed as mean ± SD. Statistical evaluations were carried out with GraphPad Prism software (v8.0). A Student’s *t*-test was applied for two-group comparisons, while one-way ANOVA was utilized for multi-group analyses. Statistical significance was defined as *p* < 0.05.

## 3. Results

### 3.1. Screening of Differentially Expressed miRNAs in T Memory Stem Cells (T_SCM_)

Our prior investigations enabled the successful isolation of four phenotypically and functionally distinct T lymphocyte subsets: naive (T_N_), stem cell-like memory (T_SCM_), central memory (T_CM_) and effector memory (T_EM_) populations, from three healthy donors through flow cytometric sorting, yielding a total of 12 T cell samples. Total RNA was extracted from these T cell samples; however, due to the limited abundance of T_SCM_, the total RNA concentration from the T_SCM_ sample of donor 2 was insufficient for subsequent library construction. High-throughput sequencing was performed on the remaining samples using the Illumina NovaSeq 6000 platform. Following data filtering, quality control and sequence alignment of the raw sequencing data, miRNA expression profiles for the four T cell subsets were successfully obtained.

**Figure 1:**
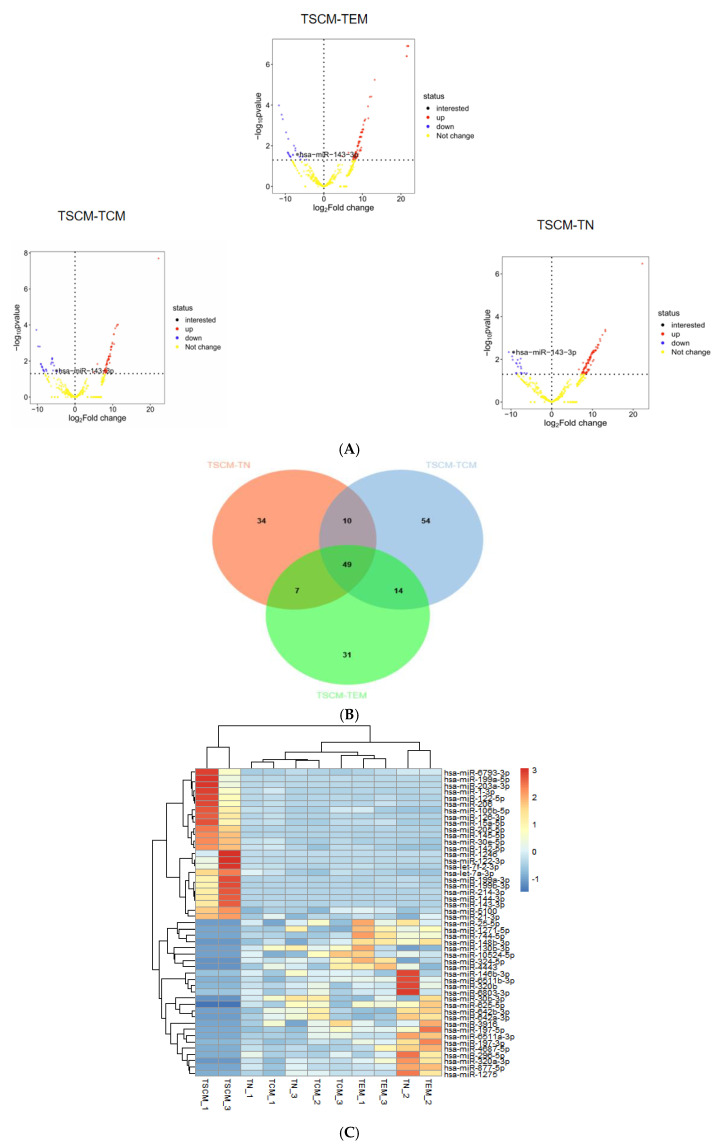
Differential miRNA expression of T_SCM_ compared to T_N_, T_CM_, and T_EM_ subsets (**A**). Volcano plots display miRNA expression differences in three comparative groups: T_SCM_ vs. T_N_, T_SCM_ vs. T_CM_ and T_SCM_ vs. T_EM_. Significant thresholds were set at *p* < 0.05 with |log2FC| > 1. The vertical axis shows −log10 *p*-values, with color gradients representing expression patterns (blue for T_SCM_-downregulated, red for T_SCM_-upregulated). The horizontal axis indicates log2FC values. miR-143-3p is highlighted in the plots. (**B**). The intersection of differentially expressed miRNAs from the comparisons of T_SCM_-T_N_, T_SCM_-T_CM_ and T_SCM_-T_EM_ was analyzed, identifying 49 miRNAs that were consistently differentially expressed in T_SCM_. In the Venn diagram, orange represents miRNAs differentially expressed in T_SCM_-T_N_, blue represents miRNAs differentially expressed in T_SCM_-T_CM_ and green represents miRNAs differentially expressed in T_SCM_-T_EM_. (**C**). The heatmap visualizes expression profiles of 49 differentially expressed miRNAs in T_SCM_ among four T cell subsets. Rows represent individual miRNAs and columns indicate samples. A color gradient reflects expression intensities (red: high; blue: low). miRNA clustering patterns are shown by the left dendrogram, with corresponding names listed at right. Sample identifiers are displayed beneath the heatmap.

To identify differentially expressed miRNAs in T_SCM_, we conducted differential expression analysis using the R programming language. The analysis focused on three specific comparisons: T_SCM_ versus T_N_, T_SCM_ versus T_CM_ and T_SCM_ versus T_EM_. The limma package was utilized for this analysis, with the latter subset in each comparison serving as the reference group. Volcano plots illustrating the differentially expressed miRNAs between T_SCM_-T_N_, T_SCM_-T_CM_ and T_SCM_-T_EM_ were generated based on *p*-values, as depicted in Figure 1A. Following the identification of differentially expressed miRNAs from these three comparisons, we performed an intersection analysis to identify 49 miRNAs that were consistently differentially expressed in T_SCM_ (Figure 1B). Subsequently, we conducted a clustering heatmap analysis of these 49 differentially expressed miRNAs in T_SCM_ across all groups using the Complex Heatmap package, as shown in Figure 1C.

### 3.2. miR-143-3p Plays a Potential Role in T-Cell Differentiation and Proliferation

Differential miRNA profiling revealed a stage-specific expression pattern of miR-143-3p during T cell differentiation. While undetectable in unstimulated naive T cells (T_N_), miR-143-3p expression was markedly upregulated during early-differentiation phases, peaking in T stem cell memory (T_SCM_) populations. Subsequent differentiation into central (T_CM_) and effector (T_EM_) memory subsets was associated with progressive downregulation of miR-143-3p, suggesting its potential regulatory role in the hierarchical differentiation process, particularly in T_SCM_ commitment.

**Figure 2:**
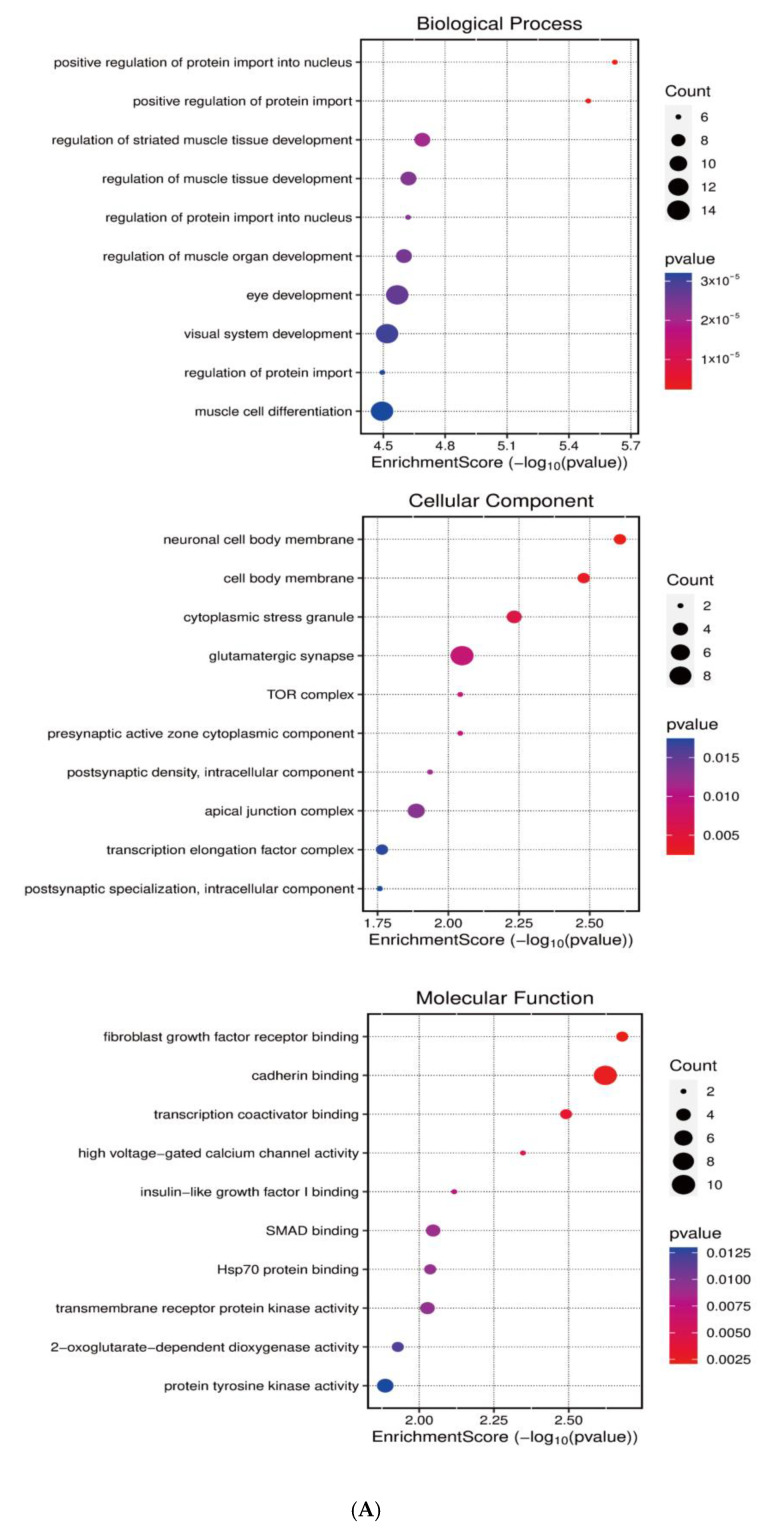

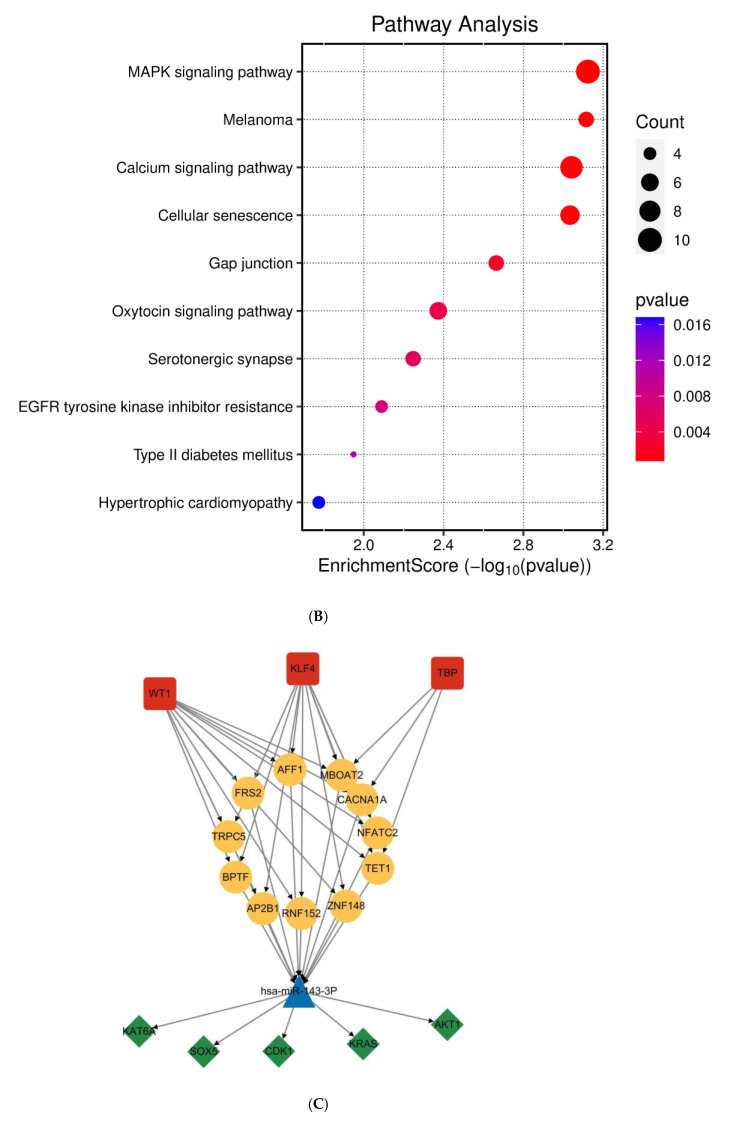

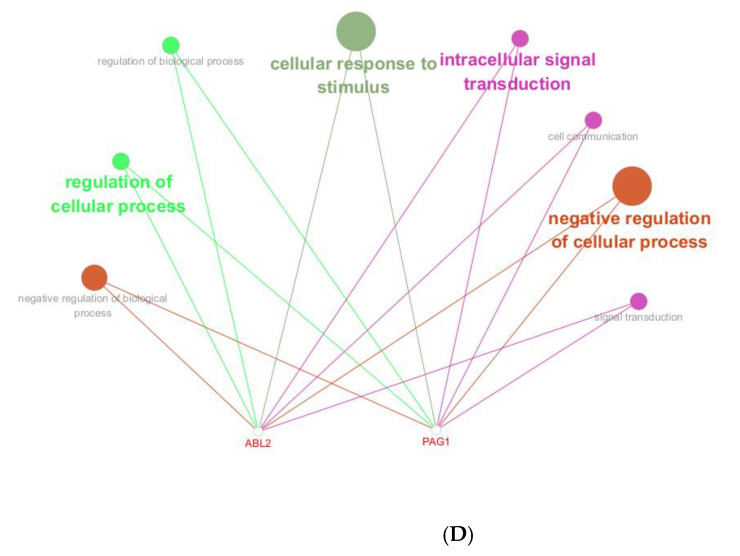
Functional annotation of miR-143-3p regulatory targets (**A**). Gene Ontology (GO) analysis of miR-143-3p targets reveals the ten most significantly enriched terms within each ontological category: cellular components, molecular functions and biological processes. (**B**). KEGG analysis reveals the top 10 enriched pathways for miR-143-3p targets. The Gene Ratio (*x*-axis) indicates enrichment degree, while pathway names are on the *y*-axis. Dot size corresponds to gene counts per pathway (larger = more genes), with color gradient reflecting *p*.adjust values (red = higher; blue = lower). (**C**). Regulatory network of miR-143-3p with three T cell differentiation-related transcription factors (TFs). Red squares: TFs; yellow circles: co-regulated targets (experimentally validated); blue triangle: miR-143-3p; green diamonds: miRNA-regulated targets. (**D**). Network visualization of two predicted targets enriched in GO biological processes.

Systematic identification of miR-143-3p targets was achieved through integrated analysis of miRWalk and StarBase datasets to investigate its regulatory role in T cell differentiation. Next, GO and KEGG enrichment analyses were subsequently conducted on the predicted target genes utilizing the clusterProfiler package in R. As shown in Figure 2A,B, KEGG pathway analysis demonstrated that miR-143-3p target genes were predominantly enriched in pathways like MAPK signaling, calcium signaling, and cellular senescence. GO analysis revealed that these genes were mainly associated with molecular functions such as positive regulation of nuclear protein import and protein translocation.

Notably, among the predicted target genes regulated by miR-143-3p, several genes, including *DDX3X*, *FOXM1*, *NCAM1* and *SKP1*, are closely associated with T-cell proliferation, survival and differentiation. Specifically, *DDX3X* has been implicated in the regulation of T-cell pyroptosis [30]. *FOXM1* plays a critical role in regulating T-cell proliferation and survival, and it has potential regulatory functions in T-cell-mediated anti-tumor immune responses [31]. *NCAM1* is essential for T-cell migration and immunological synapse formation, suggesting its influence on T-cell function through the modulation of intercellular interactions [32]. Additionally, *NCAM1* is involved in T-cell migration, activation and effector functions [9].Both MAPK and calcium-dependent signaling pathways are essential for T-cell differentiation, activation and Treg functionality [33,34]. The cumulative experimental evidence indicates that miR-143-3p potentially orchestrates T cell differentiation and expansion through transcriptional regulation of downstream target genes.

To elucidate the functional impact of miR-143-3p on T-cell differentiation and proliferation, an interaction network integrating miR-143-3p and its downstream targets was established, along with transcription factors based on experimentally validated miR-143-3p target genes. As shown in Figure 2C, the results suggest a potential impact of miR-143-3p on T-cell differentiation and proliferation. Subsequently, reverse enrichment analysis was conducted on computationally predicted miR-143-3p targets, using the web-based GeneTrail 3.2 platform, and excluded experimentally validated target genes, setting a threshold of *p* < 0.05. The results showed that *ABL2* was enriched in “regulation of metabolic process” (*p* = 4.569 × 10^−2^) and “positive regulation of metabolic process” (*p* = 7.383 × 10^−4^), while *PAG1* was enriched in “intracellular signal transduction” (*p* = 1.689 × 10^−2^). Additionally, both *ABL2* and *PAG1* were enriched in “regulation of cellular process” (*p* = 1.513 × 10^−2^). Subsequent functional enrichment analysis of *ABL2* and *PAG1* within the biological Process ontology was conducted utilizing the Cluego computational tool. We found that both *ABL2* and *PAG1* were enriched in pathways such as “Immune Response” and “Cellular Response to Intracellular Signal Transduction Stimulus”, as shown in Figure 2D. These results suggest the involvement of *ABL2* and *PAG1* in T-cell differentiation and function.

### 3.3. ABL2 and PAG1 Are Target Genes of miR-143-3p in T Cells

**Figure 3:**
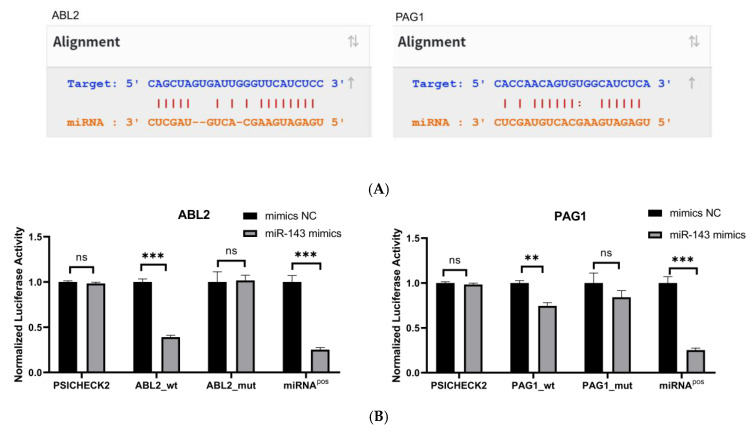

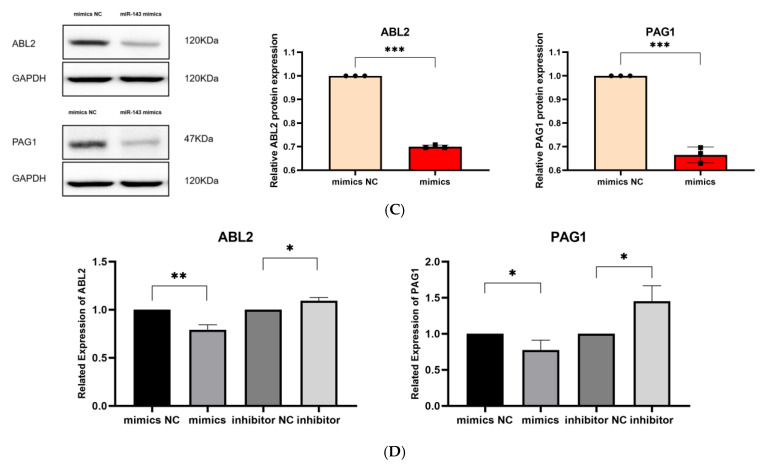
Validation of target genes. (**A**). The binding sites between miR-143-3p and the 3′-UTR of *ABL2* and *PAG1* were predicted using the StarBase database. Bidirectional arrows indicate the ranking of miR-143-3p binding sites on the target gene, with upward arrows (↑) marking the region containing the highest number of binding sites. (**B**). The dual-luciferase reporter assay (using HEK-293T cells) confirmed a direct interaction between miR-143-3p and the 3′-UTR regions of both *ABL2* and *PAG1* genes. (**C**). The protein levels of *ABL2* and *PAG1* were significantly reduced in T cells following the overexpression of miR-143-3p. (**D**). The GAPDH-normalized mRNA expression levels of *ABL2* and *PAG1* in T cells were significantly decreased following miR-143-3p overexpression. (n = 3, error bars indicate SEM; ns—not statistically significant, * *p* < 0.05, ** *p* < 0.01,*** *p* < 0.001, as determined by the *t*-test.) Prior to transfection with miR-143-3p mimics/inhibitor, all T cells had been activated through 48–60 h of stimulation.

To confirm direct binding of miR-143-3p to *ABL2*/*PAG1* 3′-UTRs, potential binding sites were predicted via StarBase (Figure 3A). Wild-type and binding-site-deficient mutant plasmids were generated. Dual-luciferase assays (Figure 3B) demonstrated significantly reduced luciferase activity (*p* < 0.01) in wild-type *ABL2*/*PAG1* groups post-miR-143-3p mimic transfection versus the mimic NC, while mutant plasmids showed no activity alteration.

We then evaluated the effect of overexpressing and inhibiting miR-143-3p on the endogenous expression of *ABL2* and *PAG1* in T cells. miR-143-3p mimics, mimic NCs, miR-143-3p inhibitor and inhibitor negative controls (inhibitor NCs) were transfected into T cells using Lipofectamine 3000 reagent. After transfection, the changes in the mRNA and protein levels of *ABL2* and *PAG1* in T cells were detected by quantitative PCR (qPCR) and Western blot (WB), respectively. As shown in Figure 3C,D, compared to the mimic NC group, the mRNA levels of *ABL2* (*p* < 0.01) and *PAG1* (*p* < 0.05) in T cells were significantly reduced following overexpression of miR-143-3p. In contrast, miR-143-3p inhibition significantly elevated *ABL2*/*PAG1* mRNA levels (*p* < 0.05). miR-143-3p overexpression markedly reduced *ABL2*/*PAG1* protein expression in T cells versus mimic NCs (*p* < 0.001). Existing evidence indicates *ABL2* (an Abl kinase member) mediates TCR-dependent signaling. The catalytic activity of Abl kinases is essential for proper T cell development and functional competence, with its deficiency impairing T cell development and partially blocking CD4+CD8+ transition [35,36]. PAG serves as a crucial inhibitory regulator in effector T cells, suppressing TCR signaling and T cell activation via membrane-associated Src kinase inactivation and Csk recruitment to the plasma membrane. Notably, PAG depletion potentiates activation responses specifically in effector T cells without affecting naïve T cells [37]. The experimental evidence demonstrates that miR-143-3p directly interacts with *ABL2* and *PAG1*, two pivotal regulators of T cell activation and differentiation programs, thereby modulating T cell differentiation through suppression of their gene expression.

### 3.4. miR-143-3p Inhibits Progressive Differentiation of T Cells

Following antigenic stimulation, naive T cells undergo activation and ultimately differentiate into effector T cells with terminal differentiation phenotypes. After the antigenic stimuli ceases, T cells undergo progressive differentiation along the T_N_ → T_SCM_ → T_CM_ → T_EM_ pathway. In addition to changes in phenotype and function, the expression of genes and transcription factors associated with T cell differentiation are also progressively upregulated or downregulated. To investigate the impact of miR-143-3p on the progressive differentiation of T cells, we transfected stimulated T cells with miR-143-3p mimics, mimic negative controls (mimic NCs), miR-143-3p inhibitor and inhibitor negative controls (inhibitor NCs) using the Lipofectamine 3000 reagent.

**Figure 4:**
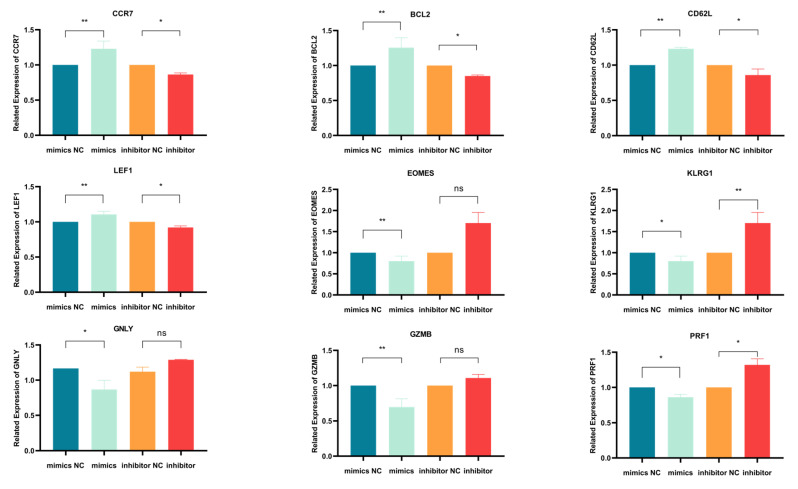
qPCR Analysis of the effects of miR-143-3p on the expression of genes and transcription factors associated with T cell differentiation. n = 3. with results expressed as mean ± SEM. Paired *t*-tests determined statistical significance (* *p* < 0.05, ** *p* < 0.01, ns—not statistically significant). Prior to transfection with miR-143-3p mimics/inhibitor, all T cells had been activated through 48–60 h of stimulation.

Using qPCR, we initially evaluated how miR-143-3p modulation affects mRNA expression of T cell differentiation markers. Figure 4 demonstrates mRNA alterations in nine genes: early-differentiation markers (CCR7 and CD62L), their transcription factor LEF1, late-differentiation marker KLRG1, its transcription factor EOMES, anti-apoptotic BCL2 and effector molecules (GZMB, PRF1 and GNLY). As illustrated in Figure 4, miR-143-3p overexpression upregulated early-differentiation markers CCR7 (*p* < 0.01) and CD62L (*p* < 0.01), their regulator LEF1 (*p* < 0.01) and BCL2 (*p* < 0.01), while downregulating late-differentiation marker KLRG1 (*p* < 0.05) and effector molecules GZMB (*p* < 0.01) and PRF1 (*p* < 0.05). Conversely, miR-143-3p inhibition reduced early-differentiation markers CCR7 (*p* < 0.05) and CD62L (*p* < 0.05) with LEF1 (*p* < 0.05), but elevated late-differentiation marker KLRG1 (*p* < 0.01) and effector molecule PRF1 (*p* < 0.05).These findings suggest that T cells overexpressing miR-143-3p exhibit gene expression profiles characteristic of early-differentiation stages, whereas T cells with inhibited miR-143-3p expression display gene expression profiles characteristic of late-differentiation stages.

Activated T cells were transfected with a (1) miR-143-3p mimic, (2) mimic NC, (3) miR-143-3p inhibitor or (4) inhibitor NC. Following RNA isolation, qPCR analysis quantified mRNA expression of early-differentiation markers (CCR7, CD62L), their transcriptional regulator LEF1, late-differentiation marker KLRG1, its transcriptional regulator EOMES and anti-apoptotic factor BCL2. Experiments were performed in triplicate (n = 3), with results expressed as mean ± SEM. Paired *t*-tests determined statistical significance (* *p* < 0.05, ** *p* < 0.01).

**Figure 5:**
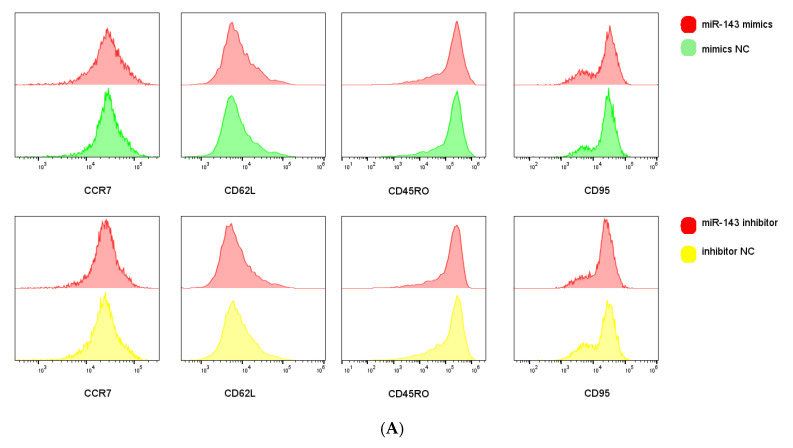

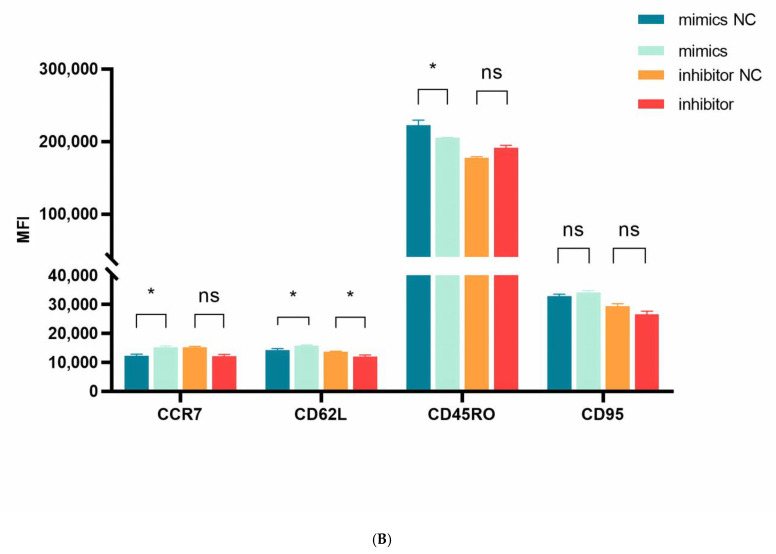

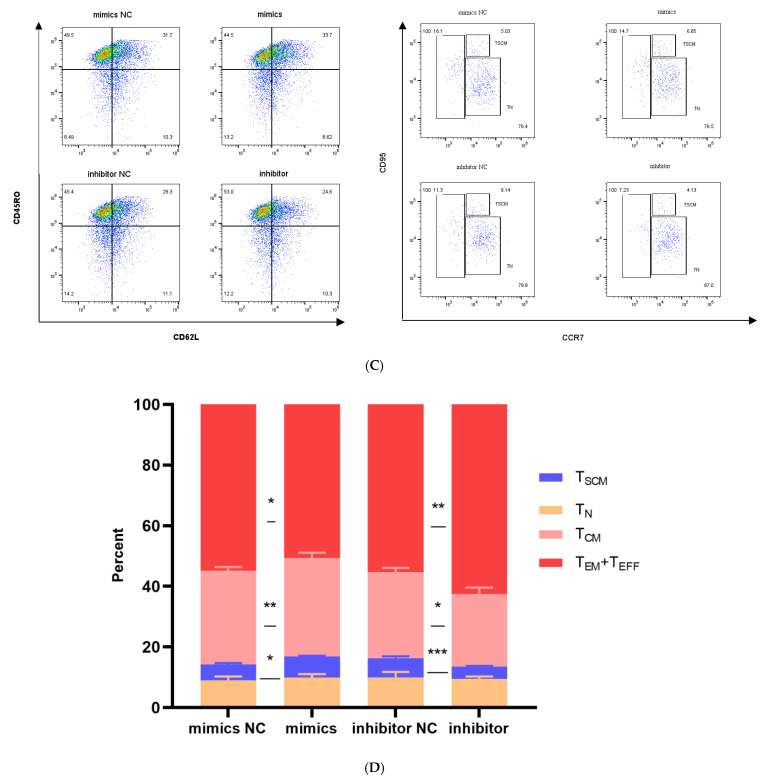
Effects of miR-143-3p on the differentiation phenotype of T Cells. Stimulated T cells were transfected with miR-143-3p mimic, mimic NC, miR-143-3p inhibitor and inhibitor NC. (**A**). Flow cytometric quantification of CCR7, CD62L, CD45RO and CD95 expression (CD3+ gated), presented as mean fluorescence intensity (MFI). (**B**). Statistical analysis of MFI values for CCR7, CD62L, CD45RO and CD95 in each group. n = 3; data are presented as the mean ± SEM. A paired *t*-test was used for statistical analysis, with ns—not statistically significant, * *p* < 0.05. (**C**). T cells (gated on CD3-positive T cells) were subdivided into subsets based on phenotypic marker expression by flow cytometry on day 5 after transfection with miR-143-3p (scatter plots). The subsets include T_N_ cells (CD3+ CD45RO− CCR7+ CD62L+ CD95−), T_SCM_ cells (CD3+ CD45RO− CCR7+ CD62L+ CD95+), T_CM_ cells (CD3+ CD45RO+ CCR7+ CD62L+), T_EM_ and T_EFF_ cells (CD3+ CD45RO+ CCR7− CD62L−). (**D**). Statistical analysis of the proportions of each T cell subset. n = 3; data are presented as the mean ± SEM. A paired *t*-test was used for statistical analysis, with * *p* < 0.05, ** *p* < 0.01 and *** *p* < 0.001. Prior to transfection with miR-143-3p mimics/inhibitor, all T cells had been activated through 48–60 h of stimulation.

We next examined miR-143-3p-mediated phenotypic alterations in T cells. Flow cytometric analysis quantified expression of early-differentiation markers (CCR7 and CD62L) and late-differentiation markers (CD45RO and CD95) in CD3+ T cells. Figure 5A,B demonstrate that miR-143-3p overexpression significantly enhanced CCR7/CD62L fluorescence intensity versus mimic NC (*p* < 0.05), but reduced CD45RO signal (*p* < 0.05). Inhibitor-mediated miR-143-3p suppression significantly decreased CD62L fluorescence (*p* < 0.05).

To elucidate miR-143-3p’s role in T cell differentiation, we stratified T cells into distinct differentiation stages using CD45RO, CCR7, CD62L and CD95 expression profiles:

T_N_ cells (CD3+ CD45RO− CCR7+ CD62L+ CD95−);

T_SCM_ cells (CD3+ CD45RO− CCR7+ CD62L+ CD95+);

T_CM_ cells (CD3+ CD45RO+ CCR7+ CD62L+);

T_EM_ and T_EFF_ cells (CD3+ CD45RO+ CCR7− CD62L−).

As shown in Figure 5C,D, compared to the mimic NC group, overexpression of miR-143-3p in T cells significantly increased the proportion of the T_SCM_ (*p* < 0.05) and T_CM_ subset (*p* < 0.01), while the proportions of the T_EM_ and T_EFF_ subsets significantly decreased (*p* < 0.05). In contrast, compared to the inhibitor NC group, inhibition of miR-143-3p expression in T cells significantly decreased the proportion of the T_SCM_ (*p* < 0.001) and T_CM_ subset (*p* < 0.05), while the proportions of the T_EM_ and T_EFF_ subsets significantly increased (*p* < 0.01).

These results confirm that overexpression of miR-143-3p promotes the differentiation of T_SCM_ and inhibits the progressive differentiation of T cells.

### 3.5. miR-143-3p Promotes T Cell Proliferation and Inhibits Effector Functions

To investigate miR-143-3p’s effects on T cell proliferation and effector functions, T cells were activated for 48–72 h followed by transfection with miR-143-3p mimics, negative control mimics (NC mimics), miR-143-3p inhibitors or negative control inhibitors (NC inhibitors) using Lipofectamine 3000.

**Figure 6:**
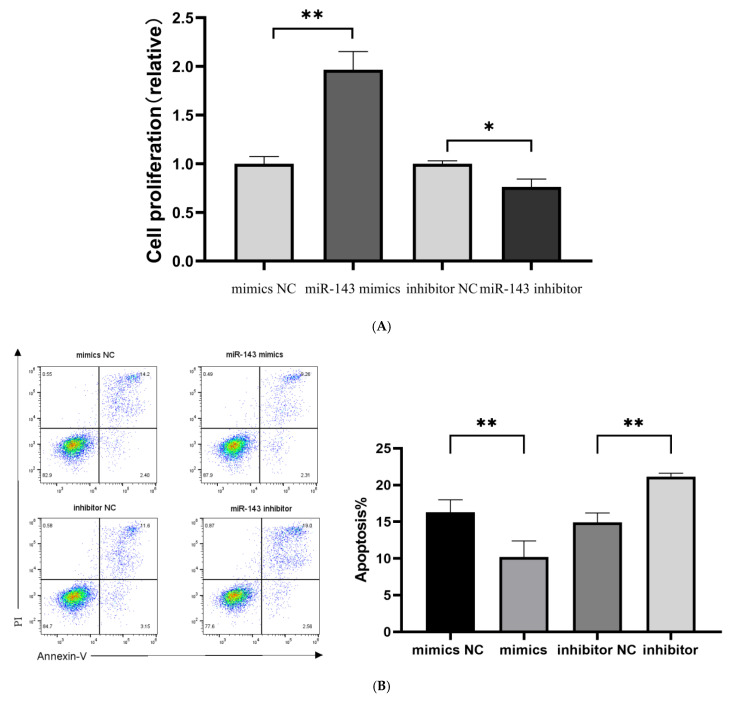

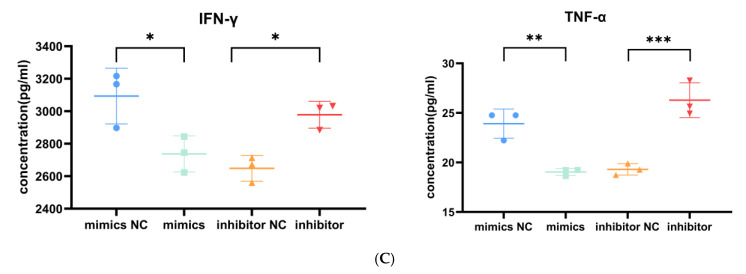
Role of miR-143-3p in T cell proliferation, apoptosis and cytokine production. Activated T cells were transfected with miR-143-3p mimics, mimic NCs, miR-143-3p inhibitor or inhibitor NCs. (**A**). CCK-8 assay revealed significantly enhanced proliferation in miR-143-3p mimic-transfected cells versus mimic NCs (*p* < 0.01), whereas inhibitor-transfected cells showed reduced proliferation compared to inhibitor NCs (*p* < 0.05). (**B**). Flow cytometric analysis of Annexin V/PI staining demonstrated significantly fewer apoptotic/dead cells (Annexin V+/PI− and Annexin V+/PI+) in mimic-treated groups (*p* < 0.01), with opposite effects observed in inhibitor-treated groups (*p* < 0.01). *X*-axis: FITC-Annexin V; *Y*-axis: PI fluorescence. (**C**). The secretion of TNF-α and IFN-γ by T cells was measured using ELISA. In the miR-143-3p overexpression group, the secretion levels of TNF-α (*p* < 0.05) and IFN-γ (*p* < 0.01) were significantly decreased. In contrast, the miR-143-3p inhibitor group showed significantly increased secretion of TNF-α (*p* < 0.01) and IFN-γ (*p* < 0.001). n = 3. Data are presented as the mean ± SEM. Statistical significance was determined using a paired *t*-test: * *p* < 0.05, ** *p* < 0.01, *** *p* < 0. 001. Prior to transfection with miR-143-3p mimics/inhibitor, all T cells had been activated through 48–60 h of stimulation.

T cell proliferative capacity was evaluated using CCK-8 reagent following a 48-h transfection period. Quantitative analysis demonstrated a marked increase in T cell counts in the miR-143-3p overexpression group compared to the mimic NCs (*p* < 0.01, Figure 6A). In contrast, miR-143-3p knockdown resulted in significantly reduced T cell numbers relative to inhibitor NC-treated cells (*p* < 0.05). These results indicate that miR-143-3p positively regulates T cell proliferation.

Five days post transfection, we evaluated the proportion of apoptotic and dead T cells using flow cytometry (Annexin V-PI double staining). As shown in Figure 6B, the number of Annexin V-positive apoptotic and dead cells in the miR-143-3p overexpression group was significantly lower than that in the mimic NC group (*p* < 0.01). In contrast, the inhibition of miR-143-3p expression resulted in a significantly higher number of Annexin V-positive apoptotic and dead cells compared to the inhibitor NC group (*p* < 0.01), indicating that miR-143-3p enhances T cell resistance to apoptosis.

T cell effector functions are characterized by cytokine production (e.g., TNF-α/IFN-γ). At 5 days post transfection, an enzyme-linked immunosorbent assay (ELISA) demonstrated that ectopic miR-143-3p expression markedly decreased IFN-γ (*p* < 0.05) and TNF-α (*p* < 0.01) production relative to mimic controls. Conversely, miR-143-3p inhibition significantly enhanced both IFN-γ (*p* < 0.05) and TNF-α (*p* < 0.001) secretion compared to inhibitor controls (Figure 6C). These findings suggest that miR-143-3p negatively regulates T cell effector functions.

## 4. Discussion

Clinical evidence from CAR-T cell immunotherapy reveals that the differentiation status of genetically engineered T cells prior to infusion critically determines their in vivo persistence and longevity, thereby modulating the durability of tumoricidal activity mediated by CAR T cells. Naive T cells (T_N_), stem cell-like memory T cells (T_SCM_) and central memory T cells (T_CM_) at early stages of progressive differentiation exhibit enhanced survival and more sustained anti-tumor immune function upon reinfusion [38,39]. Researchers have employed various strategies to increase the proportion of early-differentiated T cells, such as enriching early T cells through flow cytometry and magnetic bead sorting prior to genetic modification, optimizing culture conditions (e.g., using cytokines like IL-7 and IL-15 and low-dose anti-CD3/CD28 antibodies) to promote T cell differentiation toward a memory phenotype while inhibiting terminal differentiation [40] and blocking key signaling pathways involved in T cell progressive differentiation (e.g., inhibiting mTORC signaling) or activating Wnt-β-catenin signaling [41].

In addition to inhibiting T cell progressive differentiation, an alternative approach involves the epigenetic reprogramming of terminally differentiated T lymphocytes towards less differentiated states [42]. Notably, enforced expression of transcription factors governing early T cell differentiation (including but not limited to TCF-1, BATF-3 and FOXO-10) [43,44] has been shown to reverse the terminally differentiated state of T cells, restoring progenitor-like characteristics. Building upon this principle, our prior investigations systematically evaluated the capacity of T_N_/T_SCM_-associated transcriptional regulators to reprogram TEFF cells, culminating in the identification of an efficacious four-factor combination (BCL6, EOMES, FOXP1 and KLF7) that promotes phenotypic reversion. Additionally, knocking out diacylglycerol-kinase (DGK), conditionally deleting monocarboxylate transporter 11 (MCT11) or expressing inhibition-resistant PPAR-γ coactivator 1α (PGC-1α) has been demonstrated to induce metabolic reprogramming in T cells and influence their differentiation and function.

The molecular mechanisms regulating the progressive differentiation of T cells involve not only differentiation-associated transcription factors but also miRNAs, which play critical roles [45]. Research has shown that a single miRNA can simultaneously target multiple key genes, thereby regulating various cellular processes or signaling pathways and ultimately influencing cell differentiation [46]. With further investigation, mounting experimental evidence has established the critical modulatory functions of microRNAs in T cell differentiation and functional modulation, providing deeper insights into the mechanisms underlying T cell differentiation. For example, miR-150 directly target and suppress the transcription factor Foxo1, which induces memory T cell differentiation. Loss of miR-150 function promotes the transition of CD8+ T cells from effector to memory states [47]. miR-155 potentiates the development of both regulatory T cells (Treg) and T helper 17 (Th17) cell subsets while augmenting IL-17A secretion through *SOCS1* targeting, thereby influencing immune tolerance and autoimmune diseases [48]. Dual regulation of miR-29 and miR-130 in naïve CD8+ T cells compromises the persistence of conventional memory T cell populations while promoting the accumulation of short-lived effector T cells [49]. The miR-17~92 cluster [50] inhibits the expression of RAR-related orphan receptor α (Rora) through direct targeting, thereby suppressing the expression of T follicular helper (TFH) cell-associated genes. Conversely, overexpression of miR-17~92 promotes the generation and function of TFH cells.

In a previous study [18], we obtained miRNA expression profiles from four T cell subsets (T_N_, T_SCM_, T_CM_ and T_EM_) representing sequential differentiation stages. In the current study, we focused on screening miRNAs that are characteristically expressed in T_SCM_, which represents the earliest stage of progressive differentiation following T cell stimulation. We performed pairwise differential expression analysis between T_SCM_-T_N_, T_SCM_-T_CM_ and T_SCM_-T_EM_. After obtaining the results from these three comparisons, we conducted an intersection analysis and ranked the results based on *p*-values. Ultimately, we identified hsa-miR-143-3p as a miRNA that is characteristically upregulated in T_SCM_ and subsequently downregulated during the progressive differentiation from T_SCM_ to T_CM_ and T_EM_.

Bioinformatic prediction of miR-143-3p targets was followed by systematic functional annotation through Gene Ontology (GO) and KEGG pathway analyses. The KEGG enrichment results revealed significant involvement of miR-143-3p-regulated genes in key signaling cascades including MAPK and calcium-mediated signaling, as well as cellular senescence pathways. In the GO enrichment analysis, the target genes of miR-143-3p were mainly enriched in molecular functions including positive regulation of protein import into the nucleus and positive regulation of protein import. These findings demonstrate that miR-143-3p-regulated targets are functionally implicated in modulating multiple aspects of T cell biology, including proliferation dynamics, differentiation programs, activation states, cell cycle progression and the nuclear translocation of transcription factors. Studies have shown that upon antigen recognition by the T cell receptor (TCR) [51], the activated MAPK signaling pathway is pivotal in controlling differentiation of T cell subsets like Th1, Th2 and Th17 cells. This pathway also sustains T cell survival by modulating anti-apoptotic proteins (e.g., Bcl-2) and governs T lymphocyte migration and homing mechanisms [52,53]. The calcium-mediated signaling cascade plays a pivotal role in T cell proliferative responses and cytokine release. Of particular significance, the nuclear factor of activated T cells (NFAT) serves as a master transcriptional regulator during T cell differentiation, controlling the expression of various cytokines including IL-2, IFN-γ and IL-4 [54]. Calcium-bound calmodulin initiates calcineurin activation, leading to NFAT dephosphorylation and subsequent nuclear translocation, where it modulates genes governing T cell differentiation and functional specialization.

Moreover, we established a regulatory network comprising miR-143-3p, its target genes and associated transcription factors. Analysis demonstrated that upstream regulators of miR-143-3p and its targets are deeply implicated in T cell differentiation and maturation. These results indicate that miR-143-3p likely modulates T cell fate by controlling target gene expression, consequently altering key pathway activities (e.g., MAPK, Calcium signaling) and promoting nuclear import of transcription factors.

Bioinformatic analysis of miR-143-3p target genes revealed *ABL2* and *PAG1* as potential mediators of its effects on T cell differentiation. Luciferase reporter assays confirmed direct binding of miR-143-3p to the 3′-UTRs of both genes. Overexpression of miR-143-3p in T cells substantially reduced *ABL2* and *PAG1* expression at both mRNA and protein levels, while miR-143-3p inhibition upregulated their mRNA expression. These data establish *ABL2* and *PAG1* as direct targets of miR-143-3p-mediated regulation.

The Arg (*Abl2*-encoded) and c-Abl (*Abl1*-encoded) proteins constitute a distinct class of non-receptor tyrosine kinases. Experimental evidence indicates that TCR engagement triggers activation of endogenous Abl kinases. These kinases mediate critical immune functions through phosphorylation of key signaling molecules including ZAP-70 and LAT, which is required for (1) optimal IL-2 promoter activity, (2) TCR-dependent IL-2 production and (3) primary T cell proliferation [55].

The *PAG1* gene encodes the PAG protein, which functions as a transmembrane adaptor. PAG functions as an adaptor molecule facilitating the membrane localization of Csk, a cytoplasmic tyrosine kinase that negatively regulates T cell activation through phosphorylation-mediated inhibition [56]. Functioning as a cytoplasmic tyrosine kinase, Csk mediates the inactivation of Src-family kinases through phosphorylation of their conserved C-terminal tyrosine residues. However, because Src kinases are membrane-bound, Csk must bind membrane-associated molecules to bring it into proximity with Src kinases. As a transmembrane adaptor protein, PAG can bind Csk and is also referred to as Csk-binding protein (Cbp). Membrane-bound PAG proteins are also localized to lipid rafts, which are enriched in Src. Studies have shown that in unstimulated T cells, the tyrosine residues of PAG are phosphorylated, enabling binding to Csk. T cell receptor (TCR) engagement triggers PAG dephosphorylation, resulting in subsequent dissociation of Csk from the complex, thereby relieving Csk’s inhibitory effect on Src kinases. However, other studies suggest that although overexpression of PAG inhibits TCR signaling, knockout of the PAG gene does not result in defects in T cell development. This discrepancy arises because the inhibitory effect of PAG on TCR signaling and T cell activation primarily occurs in previously activated effector T cells, with no significant effect on naïve T cells. Furthermore, when the PAG gene is knocked out, Csk exhibits enhanced interactions with complementary binding partners (including PTPN22 and Dok adaptor proteins), which functionally cooperate with PAG to attenuate effector T cell activation [57].

Subsequently, we examined miR-143-3p’s role in T cell differentiation. qPCR analysis showed that miR-143-3p overexpression markedly enhanced the expression of early-differentiation genes (LEF1, CCR7 and CD62L) and the anti-apoptotic gene BCL2, while reducing expression of late-differentiation gene KLRG1 and effector genes (PRF1 and GZMB). Flow cytometry confirmed upregulation of early markers (CCR7 and CD62L) and downregulation of late marker CD45RO following miR-143-3p overexpression. The early-differentiated T_SCM_ and T_CM_ populations expanded significantly, whereas late-differentiated T_EM_ and T_EFF_ subsets diminished. These data indicate miR-143-3p drives T cell differentiation toward T_SCM_ while blocking terminal differentiation.

We also assessed the effects of miR-143-3p on T cell proliferation and effector functions. Overexpression of miR-143-3p enhanced T cell proliferative activity and significantly reduced the proportion of apoptotic/dead cells. Additionally, overexpression of miR-143-3p suppressed the secretion of effector cytokines TNF-α and IFN-γ. These results suggest that miR-143-3p promotes T cell proliferation while inhibiting T cell effector functions.

The data indicate that basal miR-143-3p expression in naïve T cells is relatively low. Following activation, its expression becomes upregulated and, by targeting the *ABL2* gene, it inhibits IL-2 secretion, thereby inducing T cell differentiation toward T_SCM_. During this process, the upregulation of miR-143-3p initially suppresses the expression of the PAG gene. However, due to the interactions between PTPN22 and Dok adaptors, the inhibitory effect of Csk on Src kinases is maintained. As progressive differentiation proceeds, the expression level of miR-143-3p decreases, alleviating its inhibitory effect on the PAG gene. This, in turn, promotes the membrane recruitment of Csk, enhancing Csk-mediated inhibition of Src kinases, which prevents further differentiation of effector T cells and favors the maintenance of memory T cell phenotypes.

In addition to influencing T cell differentiation, *ABL2* and *PAG1* have been identified as potential therapeutic targets in cancer. Overexpression of *ABL2* promotes tumor metastasis in various cancers (e.g., breast cancer, lung cancer) by regulating the cytoskeleton and signaling pathways [58]. *PAG1* demonstrates elevated expression profiles across multiple lymphoma subtypes and renal cell carcinomas. Functional studies reveal that *PAG1* depletion impairs the proliferative capacity, migratory potential and invasive properties of nasopharyngeal carcinoma cells [59]. Therefore, overexpression of miR-143-3p in T cells promotes their differentiation into T_SCM_, inhibits their progressive differentiation and maintains their early-differentiation phenotype, thereby enhancing the survival capacity of T cells in vivo. Furthermore, overexpression of miR-143-3p may also enhance anti-tumor effects by targeting *ABL2* and *PAG1*.

## Figures and Tables

**Table 1:** Primer sequences for qPCR analysis of T cell differentiation-related genes.

Upstream and Downstream Primers	Sequence (5′→3′)
GZMB-Forward	GTGCGGTGGCTTCCTGATAC
GZMB-Reverse	TGATGTCGTTGGAGAGTTCT
LEF1-Forward	ATGTCAACTCCAAACAAGGCA
LEF1-Reverse	CCCGGAGACAAGGGATAAAAAGT
GAPDH-Forward	TGACATCAAGAAGGTGGTGAAGCA
GAPDH-Reverse	TGTCGCTGTTGAAGTCAGAGGAC
KLRG1-Forward	CCAGAGACTCACACCTCCTTG
KLRG1-Reverse	CAGCCAGAATTGTTCCTCAGAC
BCL2-Forward	GTGGATGACTGAGTACCTGAACC
BCL2-Reverse	AGACAGCCAGGAGAAATCAAACA
GNLY-Forward	GACCTCCCCGTCCTACACA
GNLY-Reverse	AGTCCCGTGAGGTCCGTTAG
EOMES-Forward	TCGGTACGGCGTTCAATCCTT
EOMES-Reverse	TGGTCTGTGGCACGGTTCTC
CD62L-Forward	ACAACAAGAAGAACAAGGAGGACTG
CD62L-Reverse	TGTGGCAGGCGTCATCGTT
CCR7-Forward	TGTGGTCGTGGTCTTCATAGTCTT
CCR7-Reverse	CGTAGGCGATGTTGAGTTGCTTA
PRF1-Forward	CTGTGAGGAGAAGAAGAAGAAGCA
PRF1-Reverse	AGGTCGTTAATGGAGGTGTGATG

## Data Availability

Data available on request from the authors.
